# Adenosine Triphosphate/Chitin Whisker/Phenylboronic Acid-Modified Wool Fabrics with Enhanced Dyeability

**DOI:** 10.3390/ma17040893

**Published:** 2024-02-15

**Authors:** Xuemei He, Ting Zhu, Zhengkang Zhang, Guangyun Deng, Lu Cai, Haiyan Mao

**Affiliations:** Yancheng Institute of Technology, School of Textiles and Clothing, Yancheng 224051, China; hexuemeitry@163.com (X.H.);

**Keywords:** wool, chitin whisker, adenosine triphosphate, dyeing

## Abstract

Promoting the uptake of dyes is an important part of the sustainable processing of wool products. This study presents an effective modification approach to enhance the dyeability of wool fabric with adenosine triphosphate as an activator, 3-carboxyphenyl boronic acid as a ligand-binding agent, and chitin whisker as a couple agent. The structure and surface morphology of the as-prepared wool fabric was characterized in detail. Natural luteolin and acid red 1 were used to dye the modified wool fabric, and the effect of different dyeing parameters on dyeing properties was discussed. The results indicated that the modified wool gained better surface color depth (K/S) and uptake without additional agents than the untreated wool fabric. When the modified wool fabric was dyed at 45 °C with luteolin and at 60 °C with acid red 1, the dyeing processes of the two dyes on the modified wool fabrics followed the Langmuir isotherm and the pseudo-second-order kinetic model. Furthermore, the dyed modified wool fabrics possessed improved color fastness. Overall, this work offers a facile, effective, and sustainable way to improve the low-temperature dyeability of wool products.

## 1. Introduction

With increasing concerns about human health and environmental safety, the use of bio-friendly and sustainable dyeing processes has become a popular trend in the textile industry [1]. Wool has been a favorite textile fiber material for thousands of years due to its many advantages, such as excellent resilience, thermal insulation, moisture absorption, and breathability [2,3]. Wool fabrics can be dyed with acid dyes, reactive dyes, and natural dyes [4]. However, wool fabrics have poor dyeability due to the presence of the scale layer, leading to a harsh dyeing environment. For example, acid dyeing processes commonly employed for wool require high dyeing temperatures and strongly acidic conditions with the aid of a large number of auxiliary agents, resulting in reduced wet fastness as well as strength loss and severe shrinkage [5,6,7]. Natural dyes are considered to be a good alternative to synthetic dyes due to their eco-friendly, biodegradable, and less toxic nature, and additional protective function. However, some metal ion mordant agents used for enhancing natural dye uptake are mostly toxic and hazardous to both the environment and human health [8,9]. Therefore, it is crucial to improve the dyeability of wool fabrics through bio-friendly procedures to extend the availability of wool products [10,11]. In this regard, numerous efforts have been made to promote the dyeability of wool [12,13]. At present, chemical modification methods are widely recognized as highly effective approaches for enhancing both the dyeability and availability of wool products [14,15,16].

Adenosine triphosphate (ATP) consists of three phosphate groups (tri-prefix before phosphate) connected to adenosine [17]. ATP can provide readily releasable energy in the bond between the second and third phosphate groups. As a high-energy compound, it is the direct source of energy for all life activities of the organism. It plays the role of storing and transferring chemical energy and participating in the synthesis of nutrients in the organism [18]. ATP can increase the metabolic activity of cells and promote the repair and regeneration of cells. However, ATP has seldom been used in the textile field as an energy-releasing agent. Phenylboronic acid (PBA), a Lewis acid, can easily combine with 1,2- or 1,3-diols compounds to form a reversible boronate ester in an aqueous solution [19]. Therefore, PBA-containing polymers have been used as the binding domain moiety material for glucose-sensitive self-adjusting drug delivery systems, nucleotide adsorbents, sensors for sugars and glycoproteins, carriers of natural dyes, and so on [20,21,22]. Wang et al. synthesized ATP borate ester by heating nucleotide-containing substance ATP disodium with boric acid as a boron agent [23].

Chitin is a natural biological polysaccharide made of linked N-acetylglucosamine subunits, existing in crab and shrimp shells, insect exoskeletons, and fungi cell walls [10,11,24]. Recently, Chitin whisker, as a chitin derivative, has been widely used in many food and biomedical areas as a functional reinforcing material and nanofiller due to its high crystallinity and aspect ratio, non-toxicity, easy availability, and variability [25,26,27,28]. It is generally obtained by using sulfuric acid hydrolysis [29], TEMPO-oxidation [30], periodate salt oxidation, and ammonium persulfate methods [31,32,33]. Periodate can selectively oxidize the carbon−carbon bond at the 2,3-position of the polysaccharide polymer to generate bi-aldehyde groups. This property makes it highly promising for applications in carbohydrate chemistry [32]. For example, Patil et al. used the oxidation of soy flour sugar-containing aldehyde groups to enhance the mechanical strength of wool fiber [34]. Pang et al. prepared chitin whisker-complexed chitosan/dextran dialdehyde hydrogel with enhanced mechanical properties and function [35]. Ayed et al. reported chitin whiskers grafted with polyaniline as conductive reinforcing nanofillers in waterborne polymer dispersions [36]. He et al. used chitin whisker and dopamine to successfully reduce Ag nanoparticles on the surface of silk fabrics [37].

Luteolin, a naturally flavone-based polyphenol found in various plants, exhibits a wide range of pharmacological activities, including anti-inflammatory, anti-allergy, antioxidant, anti-aging, anti-cancer, antimicrobial, and antiviral properties [38]. Owing to its health-promoting and protective functionalities, the utilization of luteolin in textiles should be further explored [4]. Although luteolin has been applied for dyeing wool fabrics with antibacterial effectiveness, the current dyeing conditions require a high temperature of 80 °C, which may lead to dye instability [4].

Hence, based on the aforementioned analysis, wool fabrics were modified utilizing 3-carboxyphenyl boronic acid-coupled adenosine triphosphate as the active agent and chitin whisker as the crosslinking agent. Subsequently, the modified wool fabrics were dyed with natural pigment luteolin and synthetic dye C.I. acid red 1. The structure and surface morphology of as-prepared wool fabric were characterized using Fourier-transform infrared spectroscopy (FTIR), X-ray diffraction (XRD), thermogravimetric analysis (TGA), scanning electron microscopy (SEM), and X-ray photoelectron spectroscopy (XPS). The dyeing properties with luteolin and acid red 1 under different dyeing conditions, such as metal ion, pH value, dyeing temperature, dye concentration, and dyeing time, were discussed. Furthermore, the kinetic models and the adsorption isotherms were employed to analyze the dyeing mechanism of the modified wool fabric. Finally, an evaluation was conducted on the color fastness of the dyed wool fabrics. This work presents a feasible method to improve the low-temperature dyeability of wool fabric while reducing heat energy consumption in line with the sustainable development of dyeing processes.

## 2. Experimental

### 2.1. Materials

The wool fabric (100% pure, 180 g/m^2^) was obtained from Jiangsu Sunshine Group Co., Ltd. (Shanghai, China). Adenosine 5’-triphosphate disodium salt was supplied by Guangzhou Baiyunshan Guanghua Pharmaceutical Co., Ltd (Guangzhou, China). 3-Carboxyphenylboronic acid (98%, PBA) was purchased from Sahn Chemical Technology Co., Ltd (Shanghai, China). Sodium chloride, acetone, dimethyl sulfoxide (DMSO), and glacial acetic acid were supplied by Sinopsin Chemical Reagents Co., Ltd (Shanghai, China). Chitin (B.R.) was supplied by Aladdin Industrial Corporation (Shanghai, China). Phosphoric acid and sodium hydroxide were purchased by Jiangsu Tongsheng Chemical Reagent Co., Ltd (Yancheng, China). Luteolin was supplied by Yanghui Biotechnology Co., Ltd (Chengdu, China). Acid red 1 was purchased from Shenzhen Decai Pigment Chemical Co., Ltd (Shenzhen, China). 

### 2.2. Preparation of Chitin Whisker

Chitin whisker was prepared according to a previous work [37]. Specifically, 2 g of chitin was added to a brown conical flask containing a 0.3 M sodium iodate solution and stirred for 2 hours at 30 °C. To terminate the oxidation reaction, 20 mL of 0.1 M ethylene glycol solution was added under dark conditions. After a further incubation period of 30 min, the resulting solution was filtrated and then subjected to precipitation by adding 120 mL of acetone under dark conditions. The precipitate obtained was subsequently vacuum-filtered, washed with distilled water, and dried. Finally, the chitin whisker was obtained and denoted as WSK.

### 2.3. Modification of Wool Fabric

Firstly, the ATP/PBA solution was prepared following literature procedures with a slight modification [23]. A total of 0.2 g of adenosine triphosphate (ATP) was dissolved in 10 mL of DMSO solution, then 0.2 g of 3-carboxyphenyl boric acid (PBA) was added into the ATP solution with magnetic stirring to form the ATP/PBA DMSO solution. Then, the prepared ATP/PBA DMSO solution was added dropwise into 50 mL of 0.8% (*w/v*) chitin whisker acetic acid solution (0.5%) with magnetic stirring for 60 min to form ATP/PBA/WSK mixed solution. Finally, the weighted wool fabric (10 × 10 cm) was added into the ATP/PBA/WSK mixture solution and kept for 1 h under stirring at 30 °C in a WHY-2 constant temperature shock dyeing machine. After that, the wool fabric was taken out, washed with distilled water, and dried in the oven at 50 °C; thus, the modified wool fabric was prepared and named MWF, while the untreated wool fabric was named UWF.

### 2.4. Dyeing Procedure

The dyeing of wool fabrics was performed using the dip-dyeing method following a previously reported method [22]. The wool fabric was dyed in the sealed and conical flasks in an XW–ZDR low-noise oscillated dyeing machine (Xinwang Dyeing and Finishing Machinery Factory, Jingjiang, China). The dye concentration (luteolin and Acid red 1) was maintained at 2 mmol/L, the liquor ratio was 40: 1, and the NaCl concentration was 0.01 mol/L. The dyeing temperature was 50 °C for a hold time of 60 min without adjusting the pH value. After dyeing, the fabrics were washed in tap water and then dried in the open air. The effect of metal ions, temperature (30~90 °C), pH (1.81~10.38), time (15~270 min), and dye concentration (0.4~8 mmol/L) on dyeing properties was studied.

### 2.5. Measurement of Color Parameters

Color parameters, including color strength (K/S), L*, a*, and b* values, were measured using data color 7000 A spectrophotometer (Data Color International Ltd., UK). L*, a*, and b* represent lightness/darkness, red/green value, and blue/yellow value, respectively [39]. Each fabric sample was treated three times using the 10° standard observer under illuminant D65.

### 2.6. Measurement of Exhaustion, Dyeing Kinetics, and Adsorption Isotherm

A UV-visible spectrophotometer (TU-1901, Puchan Universal Instrument Co. LTD (Beijing, China) was used to measure the dye absorbance at maximum wavelength. The dye exhaustion (*E*%) was calculated according to Equation (1) [40]. The adsorption capacity of dye on wool fabric was calculated according to Equation (2) [41]. A_0_ and A_1_ are the absorbances of the dye bath before and after dyeing, respectively. *C*_0_ (mmol/L) is the initial concentration of dye in the solution, *C*_t_ (mmol/L) is the concentration of dye in the solution at time *t* (min), *q*_t_ (mmol/g) is adsorption capacity on wool fabric at time *t* (min), *V* (mL) is the volume of solution, and *m* (g) is the quality of the wool fabric.

(1) $$E\%=\frac{A_{0}-A_{1}}{A_{0}}\times 100$$

(2) $$q_{t}=\frac{(C_{0}-C_{t})\times V}{m}$$

The quasi-first-order kinetic mode equation fitted the adsorption kinetics curves of wool to two dyes (3) and the quasi-second-order kinetic model Equation (4) [42].

(3) $$\ln(q_{e}-q_{t})=\ln q_{e}-k_{1}t$$

(4) $$\frac{t}{q_{t}}=\frac{1}{k_{2}{q_{e}}^{2}}+\frac{t}{q_{e}}$$

where *k*_1_ is the first-order kinetic adsorption constant, *k*_2_ is the second-order adsorption constant, and *q_e_* is the dye-adsorption capacity on wool fabric at the dyeing equilibrium state.

Langmuir adsorption isotherm and Freundlich adsorption isotherm were used to study the adsorption equilibrium of solute from dyeing solution for wool fabric. Below are the linear forms of the Langmuir isotherm (Equation (5)) and Freundlich isotherm (Equation (6)) [43,44].

(5) $$\frac{C_{e}}{q_{e}}=\frac{1}{K_{L}}+\frac{a_{L}}{K_{L}}C_{e}$$

(6) $$\ln q_{e}=\ln K_{F}+\frac{1}{n}\cdot \ln C_{e}$$

where *C_e_* (mmol/L) and *q_e_* (mmol/g) are the dye concentration in the solution and the dye-adsorption capacity on wool fabric at the dyeing equilibrium state; *K_L_* and *a_L_* are the Langmuir constants related to capacity and energy of adsorption, respectively. *K_F_* is the empirical Freundlich constant or capacity factor (mmol/g), and 1/*n* is the Freundlich exponent.

### 2.7. Color Fastness Testing and Shrinkage

The light, washing, and rubbing fastness of dyed wool fabrics were tested according to GB/T 8427-2008, ISO 105-C06, and ISO 105-X12, respectively [39,45]. The shrink-resistance of treated wool samples was determined by measuring the area shrinkage. Area shrinkage was tested using a Y(B) 098D Automatic Shrinkage Testing Machine (Darong Textile Instrument Company, Wenzhou, China) according to the IWS Test Method TM31 (Washing of Wool Textile Products, including a 7 A wash cycle for relaxation shrinkage and 5 A wash cycles up to three times) and calculated according to Equation (7) [46].

(7) $$\mathrm{Area}\;\mathrm{shrinkage}(\%)=\frac{S_{0}-S_{1}}{S_{0}}\times 100\%$$

where *S*_0_ is the area of the sample before washing, and *S*_1_ is the area of the sample after washing.

### 2.8. Characterizations

A Fourier Transform Infrared spectrometer (Thermo Nicolet 6700, Mesa, Arizona, USA) was used to record the FTIR transmission spectra in the range of 400–4000 cm^−1^ using KBr disk method. The SEM images were taken using a Field Emission Scanning Electron Microscope (FEI Quanta 200 scanning electron microscope, Hillsboro, OR, USA) at an accelerating voltage of 10 kV. The thermal stability of wool fabric was analyzed using a thermogravimetric analyzer (Type STA-449C, NETZSCH Instrument Co., Ltd., Selb, Germany) in the N_2_ atmosphere. The temperature ranged from 20 to 650 °C at a scanning rate of 10 °C/min. The X-ray photoelectron spectra (XPS) were tested by the Thermo Fisher Scientific instrument (ESCALAB250Xi, Waltham, MA, USA) with the Al Kα X-ray source (1400 eV). The X-ray Diffraction (XRD) curves were determined by X’Pert3Powder diffractometer (Malvern, Netherlands PANalytical) with Cu Kα radiation in a range (2θ) between 5 and 60°. The crystallization index (CI) of wool fibers was calculated using Equation (8) [46].

(8) $$\mathrm{CI}\%=\frac{I_{I}-I_{\mathrm{II}}}{I_{I}}\times 100$$

where I_I_ is the maximum intensity around 2θ = 9°, and I_II_ is the minimum intensity near 2θ = 14°.

## 3. Results and Discussion

### 3.1. Preparation and Characterization of Modified Wool Fabric

**Scheme 1:**
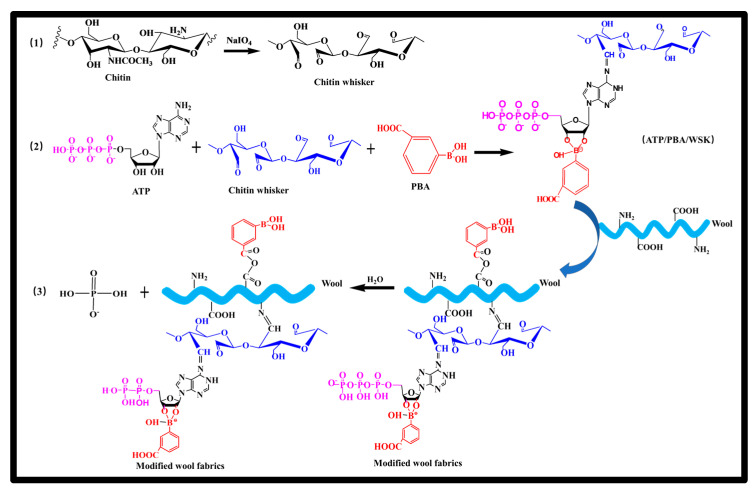
Possible reaction mechanism of modified wool fabrics.

The modification of wool fabrics was performed by immersion method in a mixing solution containing WSK, ATP, and PBA. Many crosslinking bonds, including Schiff base and boronic acid ester, were generated between the wool fiber and the active groups, such as −CHO and B−OH from ATP/PBA-activated chitin whisker [23,47]. ATP was bonded to the chitin whisker via the formation of an aldimine bond, while the dialdehyde of the chitin whisker reacted with the amino group of wool to form a Schiff base bond [48]. Additionally, the phosphate group from ATP also participated in the crosslinking process through ionic interactions with the amino group of wool fiber. Furthermore, the acid environment from the hydrolysis of ATP can cause the protonation of the amino groups on the wool fiber surface, which is favored to adsorb dye molecules. The preparation reaction mechanism is shown in Scheme 1.

**Figure 1:**
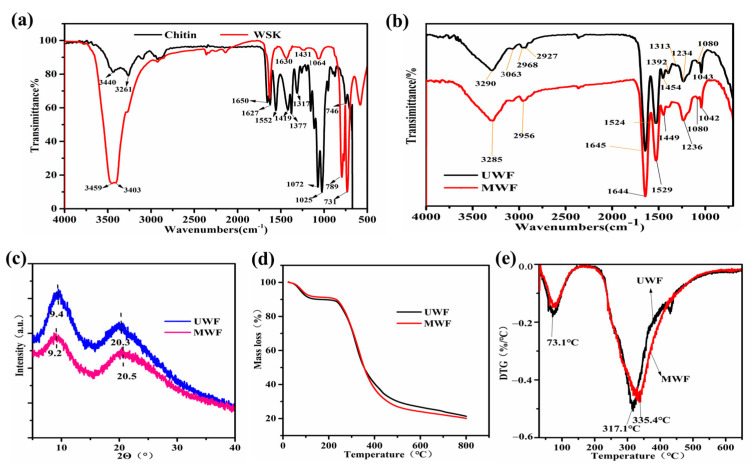
FTIR spectra of (**a**) chitin and WSK and (**b**) UWF and MWF; (**c**) XRD, (**d**) TG curves, and (**e**) DTG curves of untreated and modified wool fabrics.

The FTIR spectra of Chitin, WSK, UWF, and MWF are shown in Figure 1a,b. As revealed in Figure 1a, the chitin spectrum, the peaks for N−H and O−H stretching vibration absorption are located at 3440 and 3261 cm^−1^ [37]. The characteristic peaks for acetyl amide I and amide II are found at 1650 and 1627 cm^−1^, respectively. The peak at 1419 cm^−1^ is the acetyl methyl (CH_3_) bending vibration absorption [49]. Some peaks belonging to the skeleton vibration of the chitin pyranose ring (C−O−H) appear at 1159, 1072, 1025, and 894 cm^−1^ [50]. For chitin whisker, the peaks at 3600–3000 cm^−1^ region show thinner and more intense bands, which are attributed to the OH and NH, corresponding to the vibration of intramolecular hydrogen bonds from the aromatic ring [51,52]. The −C=O asymmetric and symmetric stretching vibration absorption peaks appeared at 1630 and 1431 cm^−1^, respectively [53]. As shown in Figure 1b, the characteristic peaks of untreated wool at 1645 cm^-1^ and 1524 cm^−1^ are, respectively, assigned to the amide I (C=O) stretching vibration band and amide II (N−H) deformation vibration band. The characteristic peaks near 3290 cm^-1^ are the N−H stretching vibration peak and the O−H stretching vibration peak [54]. The characteristic peak at 2968 cm^−1^ is the C-H stretching vibration peak, and there is a C−N stretching vibration peak at 1043 cm^−1^–1454 cm^−1^ [55]. Compared with the untreated wool fabric, the N−H stretching vibration peak, O−H stretching vibration peak, and C−H stretching vibration peak of the modified wool fabric were slightly enhanced at 3285 cm^−1^, indicating the involvement of pentose hydroxyl groups [23]. According to the spectrum of MWF, the characteristic absorption peak of borate ester is visible at 1080 cm^−1^, and the symmetrically telescopic vibration peaks of B−O are visible at 949 cm^−1^. The results indicated that the modification of wool fabric with ATP/PBA/WSK introduced more −NH_2_, −OH, and −B−OH groups on the wool surface, which contributed to the adsorption of dyes. As a whole, the reaction has no significant effect on the main chain structure of wool.

The XRD analyses of the untreated wool fabric (UWF) and modified wool fabric (MWF) are shown in Figure 1c. There are the typical characteristic peaks of wool fiber at 2θ = 9.4° and 20.4° corresponding to the α−helical and β sheet−like structures of the peptide chains in untreated wool fibers, respectively. Additionally, an observed peak at 2θ = 15.4° indicates its amorphous nature [56,57]. Following modification, a decrease in α−folded crystallinity is observed, as evidenced by the shift of the diffraction peak from 9.4° to 9.2°. The crystallization index (CI) of the modified wool fiber significantly decreases to 25.2% compared to 32.5% for the untreated wool fiber in Table 1. This reduction might be attributed to the partial breakage of the peptide or hydrogen bonds between wool fiber macromolecules caused by ATP hydrolysis and chitin whisker crosslinking [58].

The thermal stability of the UWF and MWF was analyzed by TG and DTG curves; the results are shown in Figure 1d,e. The mass-loss behavior of UWF and MWF can be divided into two stages. In the initial stage, mass loss of about 8–12% occurs within the temperature range of 30 °C to 150 °C due to the evaporation of bound and adsorbed water [59]. During this process, the unmodified wool fabric experiences a mass loss of about 11.4%, while the modified wool fabric shows a slightly lower mass loss rate at around 9.6%, indicating a decrease in water content for MWF. In the second stage, there is a weight loss of approximately 40–60% after 220 °C, mainly attributed to the decomposition of wool main chains along with disulfide bond rupture [39,56]. The mass loss of the UWF and MWF is 63.8% and 67.5%, respectively, during the whole cracking process. The temperature at which the maximum mass loss rate occurs for UWF and MWF is 317.1 °C and 335.4 °C, respectively. The increased Tmax observed in modified wool fabrics can be attributed to the presence of chitin whiskers with a high specific area and crystallinity. Furthermore, the crosslinking between fibers restricts the molecular mobility of wool polymer chains, leading to an increase in Tmax [24]. This finding aligns with previous literature suggesting that incorporating chitin whiskers into a polymer matrix slightly enhances its thermal stability [24]. At 600 °C, UWF exhibits a residual rate of 26.8%, while MWF shows a residual rate of 23.7%. Therefore, the modification process has minimal impact on the thermal stability of wool.

**Figure 2:**
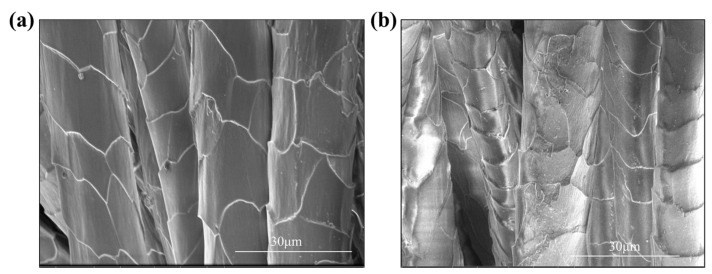
Surface morphology of (**a**) untreated wool fabric and (**b**) modified wool fabric.

The longitudinal surface morphology of wool before and after modification is depicted in Figure 2. As shown in Figure 2a, the untreated wool fiber exhibits a complete smooth scale layer structure on its surface. Figure 2b reveals slightly sharp edges of the scale layer and deposition of particles on the modified wool, indicating evident damage to the scales caused by ATP/PBA/WSK treatment. The deposited particles originate from chitin whiskers. These alterations facilitated rapid dye diffusion into the interior of wool fibers and dye uptake.

**Figure 3:**
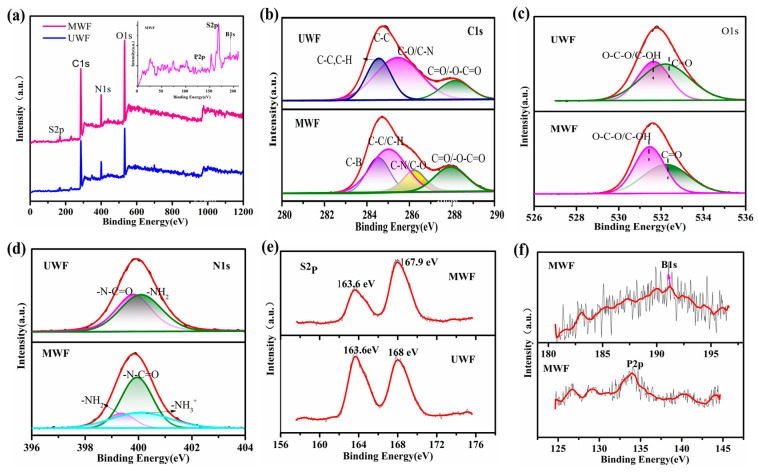
(**a**) XPS survey of wool fabrics; (**b**) C1s; (**c**) O1s; (**d**) N1s; (**e**) S2p; (**f**) B1s and P2p of modified wool.

XPS spectra were further conducted to examine the chemical composition of wool fabrics before and after modification. The Gaussian-fitted spectra are shown in Figure 3, and the elemental compositions are provided in Table 2. Figure 3a indicates that both wool samples exhibit typical elements such as C, N, O, and S on the XPS survey spectra. The peaks observed at 284.8 eV, 399.8 eV, 532.6 eV, 168 eV,133.9 eV, and 191.1 eV correspond to C1s, N1s, O1s, S2p, P2p, and B1s on the spectrum of MWF. The results were consistent with the reported literature [60]. In Figure 3b, untreated wool fabrics display three peaks located at 284.6, 285.5, and 289 eV, which can be assigned to C–C/C–H, C–N/C−O, and O−C=O/C=O, respectively. A new peak at 286.3 eV corresponding to the C–B bond is observed in MWF spectra. Compared with UWF, the peaks of C–C and C–H decreased while the C–O and O–C–O peaks increased after modification, which was attributed to the hydrolysis action of ATP and Schiff base reaction. Similarly, in Figure 3c, the total O content for MWF exhibits an increase as a result of the Schiff base reaction, consequently leading to an augmentation in the peak area of O–C–O and C–O bonds [46]. The O1s signal of UWF consists of two deconvoluted peaks, with the first peak located at 532.2 eV representing the (C=O) and the second peak situated at 531.6 eV representing the carboxylic oxygen atom (C-OH) [61]. The N1s peak at 399.8 eV of MWF exhibits a higher intensity compared to that of UWF in Figure 3d, implying a greater abundance of the N-C=O groups. Additionally, a new peak at 400.1 eV corresponding to -NH_3_^+^ was observed. In Figure 3e, the S2p spectrum of UWF can be deconvoluted into two groups: C–S–S–C signals at 163.6 eV and signals representing the oxidized sulfur species at 168 eV [61,62]. After modification, the signal at 167.9 eV exhibited an increase due to the oxidization of sulfur species induced by the presence of chitin whisker. Figure 3f illustrates the signals of B1s and P2p, providing evidence for the existence of B and P elements. Table 2 indicates that the N content increased from 11.21 to 12.48%. In comparison with UWF, there was a noticeable enhancement in both relative O/S and N/S atomic ratios, reaching values of 7.36 and 4.32, respectively. These results proved that the surface oxygen and nitrogen content increased with the introduction of ATP/PBA/WSK.

### 3.2. Surface and Mechanical Performance of Modified Wool Fabric

**Figure 4:**
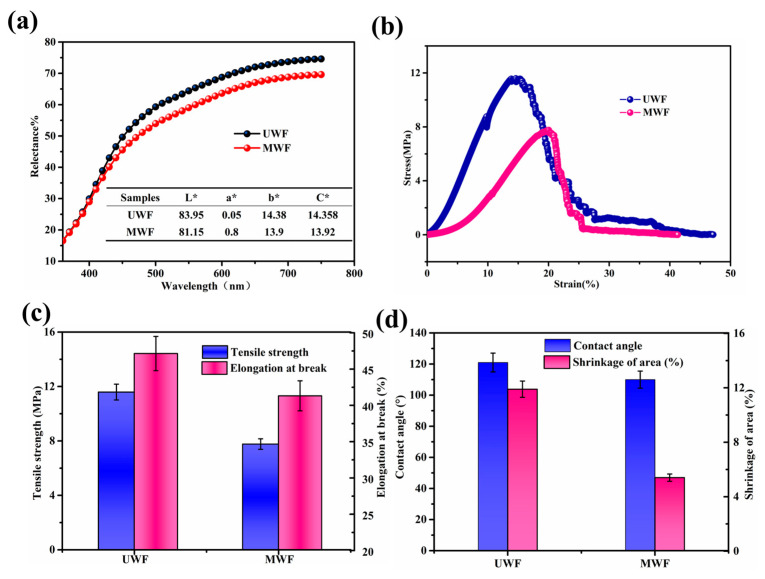
Different properties of wool fabrics before and after modification: (**a**) optical property; (**b**) stress–strain curves; (**c**) tensile strength and elongation at break; (**d**) contact angle and shrinkage of area.

The reflectance rate and color parameters, including L*, a *, and b * values, are shown in Figure 4a. The reflectance of the modified wool fabric is slightly lower than that of the untreated fabric. However, the modified wool shows similar CIE Lab color indices compared with untreated wool, meaning the modification had little effect on the appearance of the wool fabric. Figure 4b further presents typical stress–strain plots of the untreated and modified wool fibers. As seen in Figure 4b, modified wool exhibits a lower mechanical strength than unmodified wool. The elongation at break decreased from 48.8% for untreated fibers to 38.1% for modified wool in Figure 4c. This can be attributed to the hydrolysis of ATP breaking the wool main chain macromolecular. Additionally, intermolecular covalent bonding between the fibrils occurs through the Maillard reaction between aldehyde groups from the chitin whisker and amine groups of the wool molecules, leading to a decrease in tensile strain by restricting molecular movement [20]. Water contact angles of UWF and MWF were measured and shown in Figure 4d. MWF exhibited a water contact angle of 98°, which was lower than that of UWF (121°). The hydrophilicity improvement of MWF depended on the destruction of the scale layer through ATP hydrolysis and the introduction of the hydrophilic groups, including −PO_4_^−^, −COOH, and −OH, on the surface of wool via ATP/PBA/WSK treatment [23]. The enhanced wettability was beneficial to the swelling of fibers and the diffusion of dyes [46]. In Figure 4d, MWF exhibits a significantly lower area shrinkage rate of 5.4% compared to the higher value of 11.9% for UWF. During the modification process, initial ATP hydrolysis damaged the hydrophobic scales layer of wool fibers, while the crosslinking of chitin whiskers between fibers enhanced dimensional stability in water, thereby reducing the rate of area shrinkage.

### 3.3. Effect of Dyeing Conditions on the Properties of Wool Fabric

#### 3.3.1. Addition of NaCl

**Figure 5:**
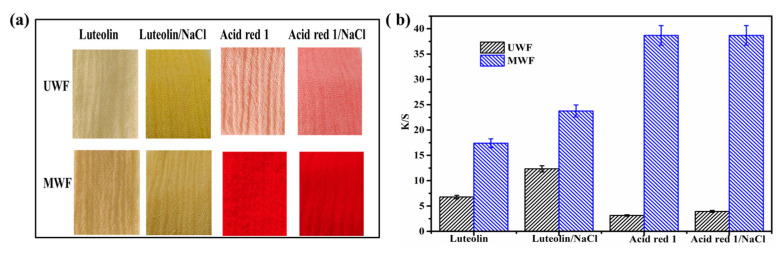
Effect of NaCl on dyeing properties of wool fabrics: (**a**) optical photographs of dyed wool; (**b**) K/S values.

**Scheme 2:**
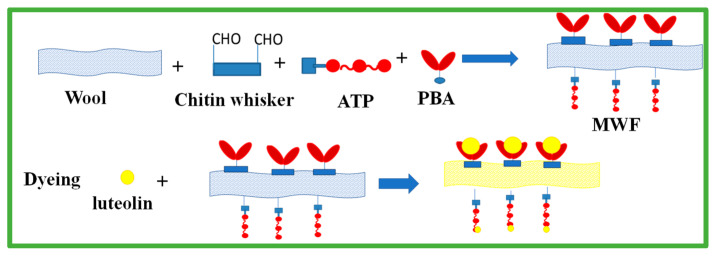
Luteolin dyeing mechanism of modified wool fabrics.

Generally, the presence of NaCl in the dye solution has an important influence on the adsorption process of the dyes. The wool dyeing process was carried out under specific conditions, including the NaCl concentration of 0.01 mol/L, dye concentration of 2 mmol/L, dyeing temperature of 50 °C, and dyeing time of 60 min. As shown in Figure 5a, the dyed MWF with luteolin and acid red 1 exhibits a notably enhanced color intensity after the addition of sodium salts. This can be attributed to the rapid adsorption of small-volume sodium ions onto the surface of wool fibers, thereby accelerating the adsorption rate for anionic dye molecules [59]. Figure 5b demonstrates that the color strength (K/S) values for the dyed MWF were markedly elevated compared to the untreated wool fiber, particularly for acid red 1. These results indicated that modification treatment could enhance the dyeability of wool fiber by facilitating energy release and hydrogen proton formation through ATP hydrolysis. Additionally, owing to its directional binding ability towards luteolin containing a 1,2− or 1,3−diols structure, the phenylboronic acid groups present in modified wool fibers readily form reversible cyclic boronic acid esters [47,63]. The proposed mechanism for dyeing modified wool fabrics is illustrated in Scheme 2. However, considering environmental concerns associated with salt usage, subsequent experiments were conducted without employing salt.

#### 3.3.2. Dyeing Temperature

**Figure 6:**
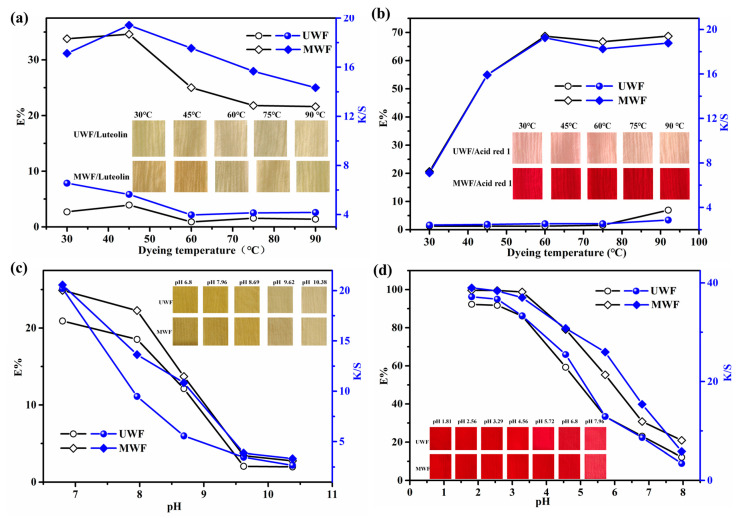
Effect of dyeing temperature and pH on dyeing properties of wool fabrics: (**a**,**c**) E% and K/S values of wool dyed with luteolin; (**b**,**d**) E% and K/S values of wool dyed with acid red 1.

The effect of the temperatures on dyeing performance was studied without adjusting the pH and adding NaCl. The exhaustion (*E%*) and the color strength (K/S) of wool fabric dyed with luteolin and acid red 1 are shown in Figure 6a and Figure 6b, respectively. The luteolin created yellowish-brown colors corresponding to the high positive values of b*, while acid red 1 exhibited redness colors. When dyed with luteolin, the K/S and dye exhaustion for MWF increased with the increase in dyeing temperatures lower than 45 °C (Figure 6a). Beyond 45 °C, the K/S and dye exhaustion decreased with the increase in dyeing temperature. This is related to the poor stability of luteolin under high temperatures, which easily causes oxidization, decomposition, and fade [64]. While dyeing using acid red 1, the K/S and dye exhaustion for both UWF and MWF increased with the increase in dyeing temperature. The results proved that higher temperatures facilitated the diffusion of molecular dyes. Surprisingly, there is a significant difference in that the dyed MWF had higher K/S and exhaustion and deeper color appearance than the dyed UWF in the same condition (Figure 6b). This can be attributed to more energy released during ATP hydrolysis, resulting in hydrogen proton formation as well as a larger number of active groups on the fiber surface, which favorably promote adsorption reactions between wool fibers and dyes. However, the change in appearance for the dyed MWF beyond 60 °C was not obvious. Therefore, considering energy-conservation concerns, it is recommended that the dyeing temperature for MWF be set at 45 °C for luteolin and at 60 °C for acid red 1.

#### 3.3.3. pH Value

The pH of the dye solution significantly influenced the dye-adsorption process. Luteolin exhibited poor solubility in acid conditions, thus limiting its pH range for dyeing to neutral or alkaline conditions. Figure 6c shows that both UWF and MWF experience a decrease in the K/S and exhaustion as the pH increases during luteolin dyeing, which can be attributed to the enhanced repulsion between the dyes and fibers under alkaline circumstances. Figure 6d demonstrates a decrease in K/S and exhaustion with increasing pH during Acid red 1 dyeing. This can be explained by the easier formation of van der Waals forces between acid red 1 and wool fibers due to the protonation of more amino groups on the fiber surface into –NH_3_^+^ under acidic conditions [65]. However, protonated amino groups are deprotonated under alkaline conditions, leading to more repulsion between electronegative groups of wool fiber and dye. Therefore, the K/S and exhaustion decreased. However, the difference between MWF and UWF was not obvious. Considering the characteristics of the two dyes, to avoid the influence of pH on the experiments, subsequent experiments were performed without adjusting the pH [59].

#### 3.3.4. Dyeing Time and Kinetic Behavior

**Figure 7:**
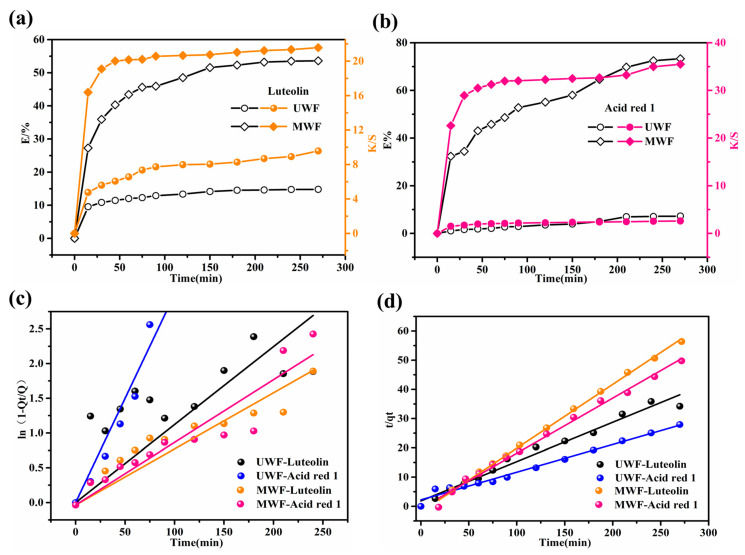
Influence of different times on dyeing performance and kinetics: (**a**) E% and K/S of dyed wool with luteolin; (**b**) E% and K/S of dyed wool with acid red 1; (**c**) quasi-first-order kinetic model fitting curves; (**d**) quasi-second-order kinetic model fitting curves.

The effect of time on the dyeing of the UWF and MWF and their adsorption kinetics were investigated, and the results are shown in Figure 7. The dyeing experiment was conducted without adjusting pH at the dye concentration of 2 mmol/L, with luteolin dyed at 45 °C and acid red 1 dyed at 60 °C. In Figure 7a and Figure 7b, it can be observed that both dyes exhibit an increasing trend in dye exhaustion (E%) and surface color strength (K/S) over a prolonged time. The adsorption speed was fast at the initial stage of adsorption, and the adsorption capacity increased rapidly. However, the adsorption sites were gradually occupied by dyes during the adsorption reaction, and the reduction in adsorption sites led to a gradual balance in the adsorption capacity. The results indicated that MWF had higher adsorption efficiency compared to UWF. The adsorption data of both dyes were fitted using the quasi-first-order kinetic model and quasi-second-order kinetic model. The results are shown in Figure 7c and Figure 7d, respectively. The kinetic parameters of wool adsorption on both dyes are shown in Table 3. Table 3 reveals that the correlation coefficients of quasi-second-order kinetic fitting curves for both dyes on MWF are remarkably high, which means the adsorption behavior of luteolin and acid red 1 on the modified wool fabric conforms to the quasi-second-order kinetic model. These results suggest that the adsorption process is chemically controlled by electrostatic action, van der Waals force, between the dyes and the modified wool fibers.

#### 3.3.5. Dye Concentration and Adsorption Isotherm

**Figure 8:**
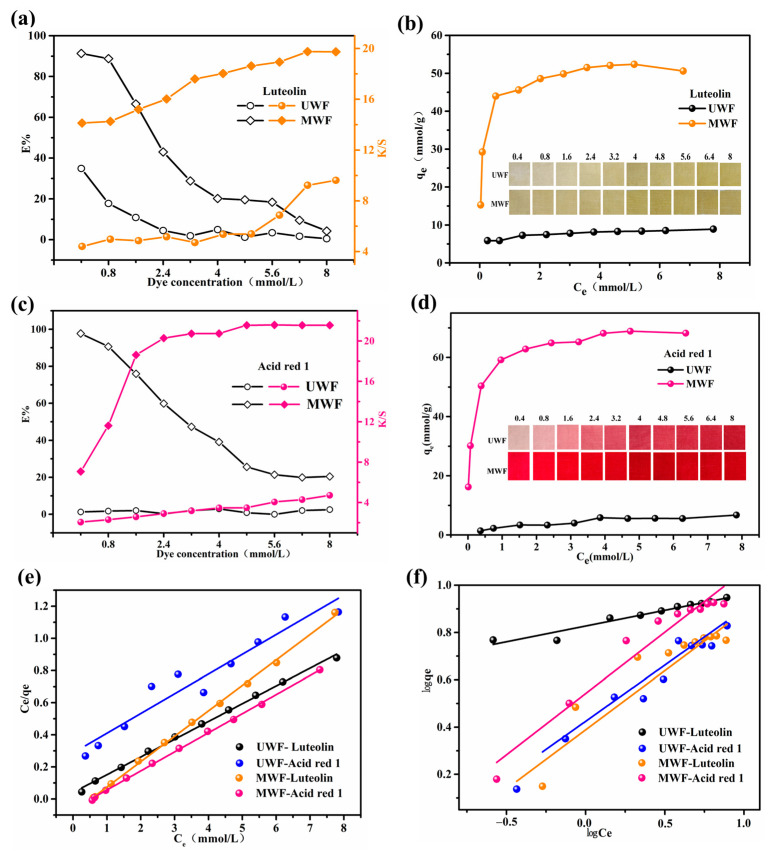
Dye concentration and fitting curves of adsorption isotherm model: (**a**) E% and K/S curves of luteolin; (**b**) adsorption capacity of luteolin; (**c**) E% and K/S curves of acid red 1; (**d**) adsorption capacity of acid red 1; (**e**) Fitting curve of Langmuir isotherm model; (**f**) Fitting curve of Freundlich isotherm model.

The effect of initial concentrations on the dyeing properties of UWF and MWF was investigated, and the results are shown in Figure 8. UWF and MWF were, respectively, dyed with luteolin at 45 °C and acid red 1 at 60 °C for 5 h without adjusting the pH. Figure 8a,c shows that the K/S and adsorption dye capacity increased with the increase in dye concentration, while the exhaustion decreased with the increasing dye concentration. Generally, the greater the dye concentration difference, the greater the dye concentration gradient, and the greater the driving force. However, when the concentration was increased to a certain extent, the adsorption capacity tended to balance because the effective adsorption sites were occupied, and no more dye groups could be introduced [59]. Figure 8b,d shows that the adsorption capacity of MWF is much higher than that of UWF for luteolin and acid red 1, respectively. This was because that modification treatment enhanced the interaction between wool fibers and dyes. To study the mechanism of the interaction between the dyes and the wool surface, the adsorption isotherms of the two dyes were obtained, and the results are shown in Figure 8e,f. Within the range of adsorption concentration, the isothermal adsorption model was fitted to the adsorption data of the two dyes, and Langmuir and Freundlich-type isotherms were used for processing, respectively.

The relevant model parameters and determination coefficients (R^2^) are shown in Table 4. According to Table 4, for MWF, the correlation coefficient of determination R^2^ = 0.9988 for luteolin and R^2^ = 0.9991 for acid red 1 is highest using the fitting of the Langmuir adsorption isotherm. The Freundlich model was not satisfactory in this study, presenting the lower correlation R^2^ = 0.8399 for luteolin and R^2^ = 0.9445 for acid red 1. This implied that the Langmuir adsorption isotherm model was more suitable for the dyeing behavior of luteolin and acid red 1 on the MWF. The results suggested that the main reaction process between MWF and both dyes was monolayer adsorption, which was controlled by electrostatic interactions and hydrogen bonding [66].

### 3.4. Fastness of Dyed Wool Fabrics

The untreated wool fiber (UWF) and modified wool fabric (MWF) were dyed with luteolin at 45 °C and acid red 1 at 60 °C using a dye concentration of 2 mmol/L without pH adjustment. Table 5 presents the washing, drying, and wet rubbing, as well as light fastness results for all dyed samples. Compared to UWF, the dyed MWF exhibited enhanced coloration fastness properties. For instance, when luteolin was used for dyeing, the light fastness was improved from level 2 to level 3, while the washing fastness of MWF reached levels 3–4 (good). Moreover, the dyed MWF with acid red 1 demonstrated excellent dry and wet rubbing resistance (levels 4–5) and very good wet rubbing resistance (level 4). These improvements can be attributed to the crosslinking between ATP/PBA activated chitin whisker and wool fibers, enhancing the affinity of wool fiber for molecular dye and easily gaining deeper shade in the dyeing process. This was favorable for improving fastness, especially for natural luteolin [56,67].

## 4. Conclusions

In summary, to achieve an effective and sustainable low-temperature dyeing effect, wool fabrics were modified using an adenosine triphosphate as an activator, 3-carboxyphenyl boronic acid as a ligand-binding agent, and chitin whisker as a couple agent. It was observed that the modification of wool fabrics resulted in enhanced dyeability for luteolin and acid red 1 due to increased energy released from ATP hydrolysis. When dyed at temperatures of 45 °C and 60 °C, respectively, without adjusting the pH, the modified wool fabrics exhibited higher K/S values and exhaustion compared to untreated wool fabrics. The adsorption process followed the Langmuir isotherm model and quasi-second-order kinetic model. The main binding mechanisms involved electro-static interactions and hydrogen bonding between the modified wool fabrics and both dyes. Furthermore, the color fastness of the modified wool fabrics showed improvement compared to untreated ones. This enhancement in dyeing properties can be attributed to the introduction of functional groups such as phenylboronic acid, amine, phosphoric acid, and hydroxyl groups on modified wool fabrics. Therefore, ATP/PBA/WSK treatment can be considered a promising and eco-friendly method for achieving low-temperature dyeing of wool products.

## Figures and Tables

**Table 1:** Crystallization index of wool samples.

Samples	I_I_	I_II_	CI (%)
UWF	5215	3523	32.5
MWF	3630	2717	25.2

**Table 2:** Content of the elements obtained by XPS.

Samples	Atomic%							
	C1s	O1s	N1s	S2p	B1s	P2p	O/S	N/S
UWF	64.98	20.59	11.21	3.22			6.39	3.48
MWF	60.35	21.26	12.48	2.89	2.66	0.37	7.36	4.32

**Table 3:** Dynamic fitting parameters of wool adsorption on two dyes.

Dyes	Samples	Quasi-First-Order Kinetic Model		Quasi-Second-Order Kinetic Model		
		*k*_1_ (1/min)	R^2^	*k*_2_ (g/mmol∙min)	R^2^	Q (×10^−2^ mmol/g)
Luteolin	UWF	0.0112	0.8209	0.00976	0.9767	7.438
	MWF	0.0188	0.9479	0.00186	0.9974	45.56
Acid red 1	UWF	0.03	0.7919	0.00403	0.9838	10.57
	MWF	0.021	0.9511	0.00106	0.9934	52.91

**Table 4:** Adsorption isotherm fitting parameters.

Dyes	Samples	Langmuir Isotherm			Freundlich Isotherm		
		*K_L_* (L/mmol)	Q _max_ (×10^−3^ mmol/g)	R^2^	*K_F_* [(mmol·g^−1^) (L·mmol^−1^)^1/n^]	1/n	R^2^
Luteolin	UWF	24.65	9.05	0.9973	6.717	0.1331	0.9418
	MWF	520.83	52.23	0.9988	40.01	0.2027	0.8399
Acid red 1	UWF	3.49	8.15	0.9255	2.446	0.4998	0.9551
	MWF	540.48	69.59	0.9991	52.22	0.2211	0.9445

**Table 5:** Color fastness of dyed wool fabrics.

Dyed Fabrics		Washing Fastness	Rubbing Fastness		Lightfastness
			Dry	Wet	
UWF	Luteolin	3	2–3	2	2
	Acid red 1	3–4	3	3–4	3–4
MWF	Luteolin	3–4	3–4	3	3
	Acid red 1	4	4–5	4	4

## Data Availability

All data generated or analyzed during this study are included in this published article.
